# Genetic and epigenetic regulation of gene expression in fetal and adult human livers

**DOI:** 10.1186/1471-2164-15-860

**Published:** 2014-10-04

**Authors:** Marc Jan Bonder, Silva Kasela, Mart Kals, Riin Tamm, Kaie Lokk, Isabel Barragan, Wim A Buurman, Patrick Deelen, Jan-Willem Greve, Maxim Ivanov, Sander S Rensen, Jana V van Vliet-Ostaptchouk, Marcel G Wolfs, Jingyuan Fu, Marten H Hofker, Cisca Wijmenga, Alexandra Zhernakova, Magnus Ingelman-Sundberg, Lude Franke, Lili Milani

**Affiliations:** University Medical Center Groningen, Department of Genetics, University of Groningen, Hanzeplein 1, 9700 RB Groningen, the Netherlands; Estonian Genome Center, University of Tartu, Riia 23 B, 51010 Tartu, Estonia; Institute of Molecular and Cell Biology, University of Tartu, Tartu, Estonia; Institute of Mathematical Statistics, University of Tartu, Tartu, Estonia; Section of Pharmacogenetics, Department of Physiology and Pharmacology, Karolinska Institutet, Stockholm, Sweden; Department of Surgery, University Hospital Maastricht and Nutrition and Toxicology Research Institute (NUTRIM), Maastricht University, Maastricht, the Netherlands; University Medical Center Groningen, Genomics Coordination Center, University of Groningen, Groningen, the Netherlands; Department of General Surgery, Atrium Medical Center Parkstad, Heerlen, the Netherlands; Department of Endocrinology, University of Groningen, University Medical Center Groningen, Groningen, the Netherlands; Department of Epidemiology, University of Groningen, University Medical Center Groningen, Unit of Genetic Epidemiology and Bioinformatics, Groningen, the Netherlands; University of Groningen, University Medical Center Groningen, Department of Pathology and Medical Biology, Molecular Genetics section, Groningen, the Netherlands

**Keywords:** eQTL, meQTL, eQTM, Gene expression, Methylation, HumanMethylation450, Liver

## Abstract

**Background:**

The liver plays a central role in the maintenance of homeostasis and health in general. However, there is substantial inter-individual variation in hepatic gene expression, and although numerous genetic factors have been identified, less is known about the epigenetic factors.

**Results:**

By analyzing the methylomes and transcriptomes of 14 fetal and 181 adult livers, we identified 657 differentially methylated genes with adult-specific expression, these genes were enriched for transcription factor binding sites of HNF1A and HNF4A. We also identified 1,000 genes specific to fetal liver, which were enriched for GATA1, STAT5A, STAT5B and YY1 binding sites. We saw strong liver-specific effects of single nucleotide polymorphisms on both methylation levels (28,447 unique CpG sites (meQTL)) and gene expression levels (526 unique genes (eQTL)), at a false discovery rate (FDR) < 0.05. Of the 526 unique eQTL associated genes, 293 correlated significantly not only with genetic variation but also with methylation levels. The tissue-specificities of these associations were analyzed in muscle, subcutaneous adipose tissue and visceral adipose tissue. We observed that meQTL were more stable between tissues than eQTL and a very strong tissue-specificity for the identified associations between CpG methylation and gene expression.

**Conclusions:**

Our analyses generated a comprehensive resource of factors involved in the regulation of hepatic gene expression, and allowed us to estimate the proportion of variation in gene expression that could be attributed to genetic and epigenetic variation, both crucial to understanding differences in drug response and the etiology of liver diseases.

**Electronic supplementary material:**

The online version of this article (doi:10.1186/1471-2164-15-860) contains supplementary material, which is available to authorized users.

## Background

The liver plays a central role in the maintenance of homeostasis and health in general. Given the substantial inter-individual variation seen in metabolism, regulation of nutrients, protein synthesis, and detoxification of xenobiotics. It is essential to have a better understanding on inter-individual variation of gene expression, methylation and genetic effects specific to liver, and on different conditions, e.g. developmental stages. These variations can affect the liver’s metabolic properties, leading to high levels of metabolites, either in the forms of lipids, proteins or xenobiotics, which can result in serious diseases or toxic side-effects. For example, several single nucleotide polymorphisms (SNPs) associated with liver function and related diseases have been identified through genome-wide association (GWA) studies [1–6]. We and others have studied how these SNPs affect liver gene expression levels by mapping expression quantitative trait loci (eQTL) [7–11], and several genetic variants that regulate genes involved in the absorption, distribution, metabolism and excretion of drugs (ADME genes) have also been identified.

Apart from genetic variation, epigenetic mechanisms (DNA methylation and histone modifications) also play an important role in regulating tissue-specific gene expression [12–14]. In particular, such mechanisms can influence the expression of hepatic ADME genes. For example, the methylation status of a CpG island in exon 2 of *CYP1A2* was shown to correlate with interindividual differences in the expression of this gene in human livers [15]. Given that CYP1A2 is an important drug-metabolizing enzyme, those factors that influence its epigenetic state may also contribute to the individual drug response. Interestingly, the epigenetic state of ADME genes, at least in rodent livers, can change in response to xenobiotic exposure [16, 17], thus opening the perspective for epigenetics-mediated drug-drug interactions. More examples on epigenetic regulation of ADME genes have been reviewed by Kacevska et al. [18]. However, the majority of such data come from studies of epigenetic alterations observed either in tumors, or in cell lines treated with DNA demethylating agents. So far it is not clear, to which extent such cancer-related or experimentally induced epigenetic alterations correspond to the natural epigenetic variability in human livers. Hence, it is essential to include epigenetic variation when studying the regulation of hepatic gene expression, to further explain the causes of differences in drug response and the etiology of diseases associated with liver function.

**Figure 1:**
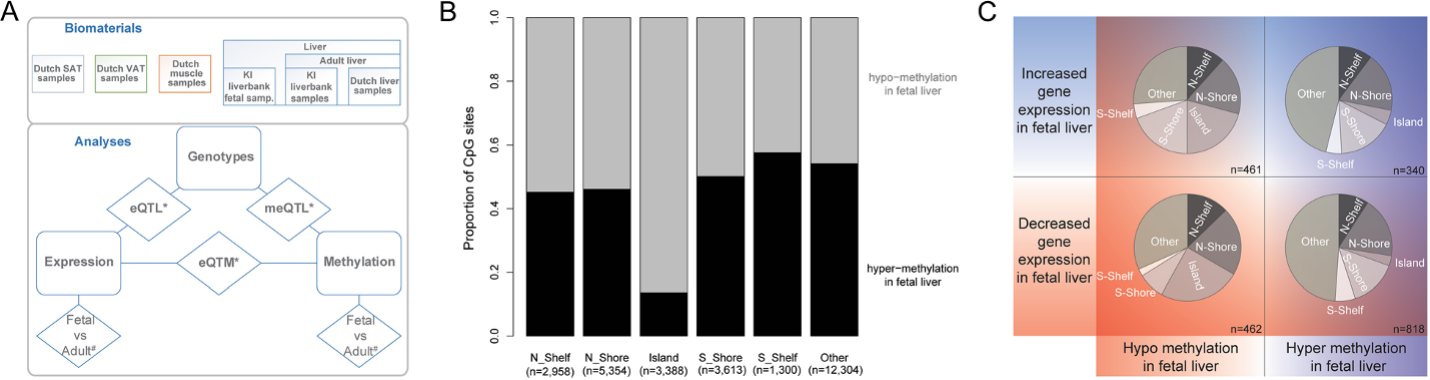
**Study design and distribution of CpG sites. (A)** Study design describing the investigated biomaterials and analyses performed. *) conservation compared across tissues; #) compared in fetal vs adult livers. **(B)** Distribution of the location of differentially methylated CpG sites between fetal and adult livers. The bar plot shows the percentage of differentially methylated CpG sites (y-axis) that are hypermethylated (black bars) or hypomethylated (grey bars) in fetal livers compared to adult livers in CpG islands, shores, shelves and other regions of the genome. **(C)** Distribution of differentially expressed and methylated genes depending on the relation to CpG islands. Pie charts illustrating the distribution of CpG island regions in case of significant increased or decreased gene expression and significant hyper- or hypomethylation.

Here we present a comprehensive survey of the methylome and transcriptome of the human liver (Figure 1A). First, we addressed the regulation of gene expression in the developing human liver by comparing genome-wide expression and methylation levels in 96 adult and 14 fetal livers from the Karolinska Liver Bank. Then we used genetic, epigenetic and gene expression data from the adults, along with an extra cohort of 85 Dutch adult liver samples to investigate the regulation of gene expression in the human liver. Finally, we explored the tissue specificity of the identified associations between SNPs, methylation and expression in other tissues from the Dutch adult samples.

## Results

### Developmental regulation of hepatic gene expression

#### The epigenome of the developing human liver

We first compared the epigenomes of 8- to 21-week-old fetal livers with adult livers. We assessed the methylation levels of 366,074 variable CpG sites and found 28,917 CpG sites (annotated to 12,619 unique genes) that showed a significant difference (absolute mean beta value difference > 0.2, FDR < 0.05) between fetal and adult liver tissue (see Supplementary Online Methods in the Additional files 1 and 2). Although the number of hypomethylated CpG sites in fetal liver (53.4%) was similar to the number of hypermethylated sites (46.6%) in this cohort, we observed an age-specific association between the genomic location of CpG sites and whether they were hypo- or hypermethylated (chi-squared test p-value < 2.2 × 10^-16^). In fetal livers, the majority (86%) of the differentially methylated CpG sites that are located within CpG islands (CGI) were hypomethylated, whereas this was not the case for CpG sites outside CGIs, where roughly 50% of the CpG sites were either hypo- or hypermethylated in fetal livers (Figure 1B). This is particularly interesting because in both adult and fetal livers, close to 80% of the CpG sites within CGI are not methylated, with > 95% overlap between the two age groups. Accordingly, the CpG sites within CGIs that were hypomethylated in the fetal livers mostly had intermediate methylation levels in the adult liver samples (Additional file 3).

To explore the functions of the genes that were differentially methylated in fetal liver compared to adult liver, we used the GREAT pathway tool [19]. The CpG sites that were hypomethylated in the adult livers and hypermethylated in fetal liver were strongly enriched for metabolic pathways, such as the steroid metabolic process the regulation of lipid metabolic processes, regulation of generation of precursor metabolites and energy, and regulation of glycolysis (all with p-values < 1.15 × 10^-44^) (Table 1A). However, the genes that were associated with hypomethylated CpG sites in the fetal samples were strongly enriched for pathways of insulin receptor signaling, regulation of glycogen synthase activity, differentiation processes, and developmental functions (Table 1B).

**Table 1:** **Gene Ontology analysis of differentially methylated genes in fetal versus adult livers**

A. Top 10 biological processes associated with hypomethylated genes in adult livers			
Term Name	P-value	Fold enrich. ^1^	Obs. regions ^2^
Steroid metabolic process	2.77E-52	2.03	558
Regulation of lipid metabolic process	4.42E-51	2.06	528
Regulation of generation of precursor metabolites and energy	5.62E-48	3.24	216
Regulation of glycolysis	1.15E-44	5.21	116
Sterol metabolic process	4.28E-44	2.56	288
Positive regulation of lipid metabolic process	3.52E-43	2.48	300
Regulation of cellular carbohydrate catabolic process	3.26E-42	4.22	136
Regulation of lipid transport	3.88E-42	3.54	168
Cholesterol metabolic process	3.71E-40	2.51	271
Regulation of cellular ketone metabolic process	4.25E-39	2.11	381
**B. Top 10 biological processes associated with hypomethylated genes in fetal livers**			
**Term Name**	**P-value**	**Fold enrich.** ^**1**^	**Obs. regions** ^**2**^
Insulin receptor signalling pathway	1.74E-130	77.00	88
Positive regulation of glycogen (starch) synthase activity	1.69E-105	37.19	90
Anterior/posterior pattern specification	1.27E-96	2.26	813
Regulation of gene expression by genetic imprinting	2.36E-92	10.52	143
Regulation of glycogen (starch) synthase activity	3.97E-81	19.01	91
Genetic imprinting	3.54E-69	6.66	147
Response to estrogen stimulus	4.52E-69	2.14	657
Positive regulation of insulin receptor signalling pathway	9.60E-68	11.64	98
Positive regulation of cell cycle	2.83E-61	2.51	421
Luteinizing hormone secretion	4.18E-61	11.24	90

^1^Fold enrichment **-** fold enrichment of number of genomic regions in the test set with the annotation.

^2^Observed region hits - actual number of genomic regions in the test set with the annotation.

#### The transcriptome of the developing liver

Comparison of gene expression levels between the fetal and adult liver samples yielded 3,284 differentially expressed probes (absolute log_2_-fold change > 1.0, FDR < 0.05, Additional file 4). Pathway analysis, using Gene Network [20], confirmed that 1,396 genes with higher expression in the adult livers were strongly enriched for metabolic functions like monocarboxylic acid, steroid and bile acid metabolic processes, as well as the response to xenobiotic process (Table 2A). In contrast, 1,277 genes that were highly expressed in fetal tissue were associated with regulating organelle organization, chromosome organization, and tetrapyrrole (e.g. hemoglobin) biosynthetic processes (Table 2B). These observations are in line with the fetal development, which is characterized by tissue differentiation and growth and by the fact that the liver is predominantly a hematopoietic organ during this period [21].

**Table 2:** **Gene Ontology analysis of differentially expressed genes in fetal versus adult liver**

A. Top 10 biological processes associated with hyperexpressed genes in adult livers		
Term	P-value	Nr of genes
Monocarboxylic acid metabolic process	2.80E-205	347
Lipid localization	8.26E-202	201
Lipid transport	1.99E-197	180
Steroid metabolic process	2.91E-196	257
Bile acid metabolic process	1.13E-192	40
Response to xenobiotic stimulus	9.88E-184	114
Cellular response to xenobiotic stimulus	9.88E-184	114
Xenobiotic metabolic process	6.72E-183	113
Bile acid biosynthetic process	6.23E-178	23
Response to glucocorticoid stimulus	2.61E-173	131
**B. Top 10 biological processes associated with hyperexpressed genes in fetal livers**		
**Term**	**P-value**	**Nr of genes**
Negative regulation of organelle organization	3.93E-162	138
Regulation of organelle organization	1.73E-132	370
Negative regulation of cellular component organization	3.40E-127	265
Regulation of chromosome organization	1.61E-114	72
Porphyrin-containing compound biosynthetic process	1.84E-112	34
Tetrapyrrole biosynthetic process	1.84E-112	34
Negative regulation of chromosome organization	6.05E-108	29
Chromatin assembly or disassembly	8.76E-106	128
Pigment biosynthetic process	1.73E-103	53
G1 phase	6.25E-102	36

#### Orchestration of epigenetics and transcriptomics in regulating liver development

We found 1,655 genes that showed both differential expression and differential methylation in adult vs. fetal livers (Additional file 5). More specifically, 657 genes were linked to probes with higher expression levels in adults, and 1,000 genes linked to probes that were more highly expressed in fetal livers (with an overlap of two genes). As expected, these genes are even more significantly enriched for developmental stage-specific functions, such as drug response for the adult cohort (p-value 4.0 × 10^-131^) and liver development for the fetal cohort (p-value 6.0 × 10^-90^). In the majority of the genes with more than one detection probe, the differences in expression levels were very similar between fetal and adult livers. However, in two genes (*TGM2* and *INS-IGF2*), one of the probes was more highly expressed in fetal livers, while the other probe reflected higher expression in adult livers. The location of the differentially methylated CpG sites differed significantly in relation to CGIs, depending on the expression and methylation differences between fetal and adult livers (chi-squared test p-value < 2.2 × 10^-16^, Figure 1C): for genes with a lower expression in fetal livers, the hypomethylated CpG sites more often map within CpG islands, shores and shelves, while the hypermethylated CpG sites map further away from CGI regions.

**Figure 2:**
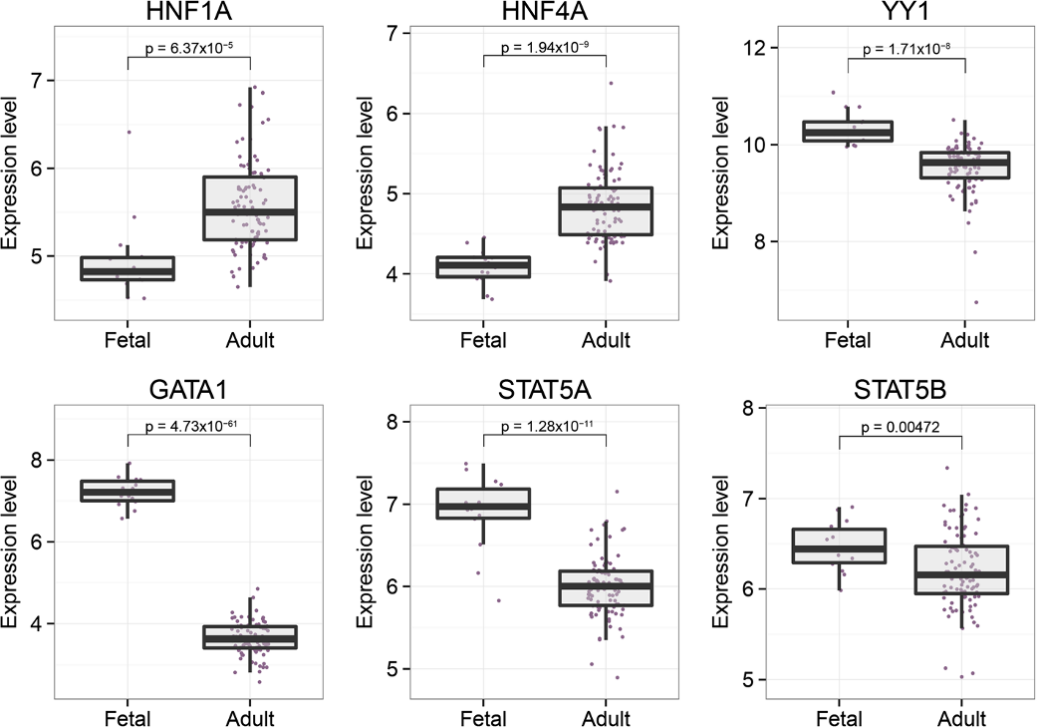
**Expression levels of transcription factors in fetal and adult livers.** Box plots of the log_2_ transformed expression levels (y-axis) are shown for the adult and fetal liver samples (x-axis). The transcripts for HNF1A and HNF4A were expressed at significantly higher levels in the adult livers, while YY1, GATA1, STAT5A and STAT5B were expressed at higher levels in the fetal livers.

Regions within 2 kb of the transcription start site (TSS) of the 1,655 genes are enriched for binding sequences of transcription factors essential for the development or function of the liver, specifically HNF4A (adjusted p-value = 2 × 10^-73^) and HNF1A (adj. p-value = 6 × 10^-38^); hematopoietic transcription factors GATA1 (adj. p-value = 8 × 10^-36^), STAT5A (adj. p-value = 2 × 10^-43^), and STAT5B (adj. p-value = 1 × 10^-49^); and YY1 (adj. p-value = 2 × 10^-36^), which plays a fundamental role in embryogenesis and differentiation. We therefore investigated the expression levels of these transcription factors and observed that transcripts for the *HNF1A* and *HNF4A* genes were more highly expressed in adult livers, and *GATA1, STAT5A, STAT5B* and *YY1* were all more highly expressed in fetal livers (Figure 2, Additional file 6).

Table 3 lists the 20 genes with the largest differences in expression and methylation, clearly illustrating the fetal-specific expression of genes involved in differentiation and hematopoiesis (e.g. *DLK1, HBZ, HBM, AHSP, EPB42* and *NFE2*), and the adult-specific expression of genes involved in drug metabolism, catabolism and other biosynthesis processes. *CYP2E1* and *CYP2C8* are the cytochrome P450 (CYP) genes; these show the most significant difference in expression levels between fetal and adult liver, with an approximately 7-fold higher expression level in adult liver.

**Table 3:** **Top 20 genes with largest difference in expression and differential methylation between fetal and adult livers**

Gene	Median expression		logFC	Adj p-value (FDR)	Mean beta value		Beta value difference	Adj p-value (FDR)
	Adult	Fetal			Adult	Fetal		
*DLK1*	3.27	12.64	9.15	3.55E-46	0.42	0.63	0.22	3.19E-36
*HBZ*	3.23	12.35	9.07	1.82E-45	0.8	0.55	-0.25	4.02E-18
*HBM*	3.01	12.52	9.03	4.27E-42	0.42	0.21	-0.21	2.55E-11
*AHSP*	4.37	13.26	8.46	2.66E-42	0.74	0.41	-0.33	1.43E-34
*EPB42*	3.13	11.25	8.19	2.26E-48	0.89	0.47	-0.42	3.84E-64
*CYP2E1*	13.42	4.1	-7.64	8.63E-36	0.51	0.88	0.36	1.10E-41
*HBE1*	2.87	10.66	7.63	4.10E-48	0.76	0.49	-0.27	1.41E-34
*CRP*	13.33	4.33	-7.27	7.71E-34	0.53	0.89	0.36	2.99E-42
*C9*	11.91	3.59	-7.18	3.98E-39	0.48	0.84	0.36	8.73E-39
*APCS*	12.69	4.39	-7	5.95E-40	0.45	0.88	0.43	7.26E-48
*SLC4A1*	4.04	11.3	6.96	1.46E-61	0.75	0.4	-0.35	2.14E-42
*NNMT*	11.06	3.44	-6.88	4.10E-40	0.36	0.84	0.48	7.10E-45
*CYP2C8*	12.9	4.83	-6.85	3.42E-31	0.59	0.88	0.29	2.57E-36
*AQP9*	11.51	3.47	-6.81	1.26E-33	0.39	0.84	0.45	8.23E-45
*NFE2*	4.1	11.03	6.8	2.29E-47	0.82	0.45	-0.36	3.02E-41
*ADH1C*	11.93	3.92	-6.69	1.96E-24	0.42	0.81	0.39	8.06E-38
*MYL4*	3.88	10.77	6.65	6.78E-60	0.88	0.4	-0.48	1.90E-62
*C3P1*	11.36	3.81	-6.63	2.34E-37	0.73	0.24	-0.49	1.56E-47
*RHAG*	3.35	10.22	6.56	1.50E-46	0.81	0.49	-0.31	1.64E-47
*HSD17B6*	12.12	4.61	-6.31	4.88E-29	0.89	0.66	-0.22	1.48E-41

### Genetic and epigenetic effects on inter-individual variability in gene expression

#### Correlation in DNA methylation and gene expression

**Figure 3:**
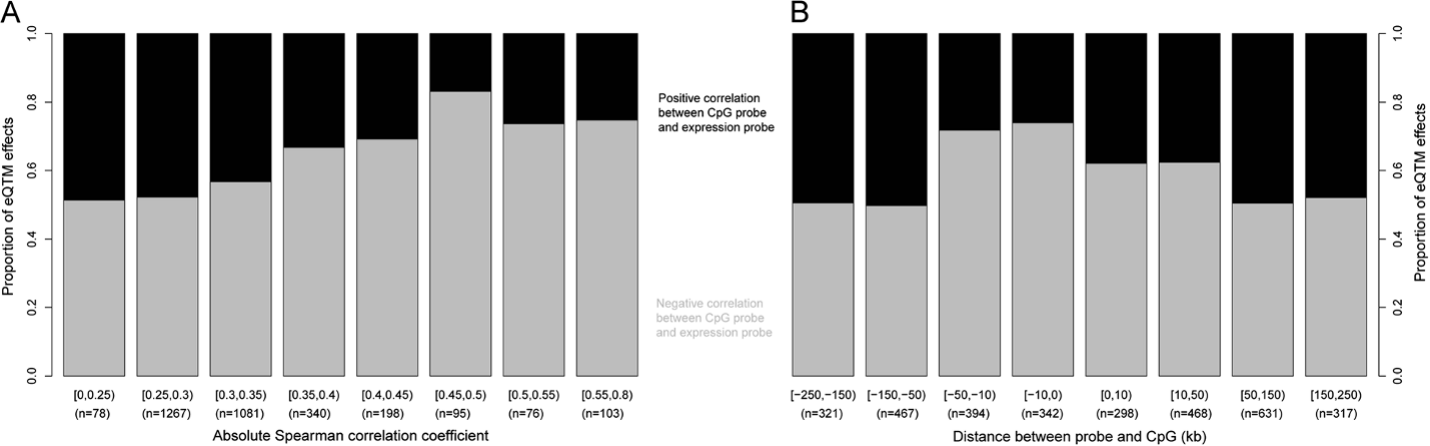
**Distribution of the direction of the expression and methylation correlation coefficient. (A)** Proportion of eQTM effects (y-axis) grouped by the absolute Spearman correlation coefficient. Grey and black colors represent negative and positive correlation between expression probe and methylation CpG site, respectively. **(B)** Proportion of eQTM effects (y-axis) grouped by the distance between expression probe and CpG site in kilobase pair (kb). Grey and black colors represent negative and positive correlation between expression probe and methylation CpG site, respectively.

We next assessed whether DNA methylation is correlated to gene expression levels in the adult samples. We combined data from the Karolinska Liver Bank and Dutch liver samples (total number of samples with expression and methylation data = 158) and compared expression probes with CpG sites that map within 250 kb of these probes. We did not include the fetal samples due to the large developmental differences reported above, and we estimated that the fetal samples would not add any considerable statistical power for the analyses. We identified 3,238 significant methylation-expression associations (eQTMs, Additional file 7), comprising 1,988 unique expression probes (in 1,798 genes) and 2,980 CpG sites (reflecting 2,057 unique genes), with a permutation p-value < 0.05. As expected, there are more eQTMs with a negative correlation between expression levels and CpG methylation levels (58.4%), irrespective of the CpG site location in relation to CpG islands. Furthermore, for CpG sites with strong correlation between expression and methylation levels, and/or within 50 kb of the expression probes, we observed an overrepresentation of negative correlations (chi-squared test p-value < 2.2 × 10^-16^, Figure 3).

#### Regulation of gene expression by genetic polymorphisms

We next explored the effects of genetic variation on liver gene expression levels. Expression quantitative trait locus (eQTL) mapping in the adult livers (meta-analysis of the two cohorts, combined number of samples with expression and genotype data = 171) yielded a total of 47,168 significant SNP-probe pair correlations (FDR < 0.05), representing 751 unique genes (Additional file 8). The eQTL probes are significantly enriched for liver-specific genes (area under the curve (AUC) 0.67, p-value 4 × 10^-57^, as reported by Gene Network) and are strongly enriched for genes encoding drug-metabolizing enzymes (p-value 2.0 × 10^-19^). We compared our results with reported liver *cis*-eQTLs [7–10, 22] and observed that we could replicate 667 reported eQTL genes, however we also identified 84 new eQTL genes (Additional file 8).

#### Influence of genetic variation on DNA methylation

We investigated the effects of SNPs on CpG methylation (meQTL) in adult liver samples (meta-analysis, combined number of samples with methylation and genotype data =161). In total we found significant *cis*-meQTL for 28,447 unique methylation probes (FDR < 0.05, mapping to 12,054 unique genes), reflecting 1,477,126 different SNP-CpG site combinations. In contrast to the eQTL, we did not observe any enrichment of liver functions for these 12,054 meQTL associated genes. Looking further into the SNPs affecting DNA methylation and gene expression, we identified 215 unique genes and 10,432 unique SNPs associated with both an eQTL and meQTL, resulting in a total of 30,644 overlapping QTL effects. Interestingly, for most of the 215 genes (69.3%) influenced by both an eQTL and meQTL we observed an opposite effect direction, i.e. the same genotype was associated with higher methylation levels and lower expression levels, or *vice versa* (Additional file 9). This effect is strongest in the CpG islands and CpG island shores, where it occurs in more than 75% of the cases (Additional file 10).

#### Contribution of genetic variants and DNA methylation to variation in hepatic gene expression

Once we had identified eQTL and eQTMs, we ascertained to what extent SNPs and DNA methylation could jointly explain the variation in liver gene expression levels. We selected 293 expression probes (reflecting 274 unique genes) that had both a significant *cis*-eQTL and significant eQTM effect. We then tested four different linear models (see Supplementary Online Methods in the Additional file 1) to assess the proportion of variation in gene expression that could be explained. For 83% of these 293 expression probes, most of the expression variation was explained by a SNP (Additional file 11), whereas for the remaining 17% the expression variation was most strongly explained by a specific CpG site. For the latter cases, we observed that these expression-associated CpG sites were likely to have a meQTL effect (chi-squared p-value = 0.035). As expected, when we combined the SNP genotype and CpG site methylation levels, we could explain more of the expression variation than by using either SNP or methylation levels alone. Given the correlations between genotypes and methylation levels, we also estimated the unique contributions of the two on gene expression levels (Additional file 12). Overall, SNP genotypes uniquely explain a greater proportion of the variation in gene expression (median 0.1, standard deviation 0.122) than methylation levels (median 0.029, standard deviation 0.049). The SNPs and CpG sites with particularly high correlations with the expression levels were generally closer to the transcription start site of the corresponding genes (Additional file 13).

The contributions of SNPs and DNA methylation levels to the proportion of variation explained in gene expression levels are illustrated in Additional file 14 and Table 4 by 16 unique ADME genes that had both significant eQTL and eQTMs. The ADME gene list was extracted from http://www.pharmaADME.org. For the *GSTT1, GSTM1, UGT1A1, GST01* and *PON1* genes, DNA methylation explains a larger proportion of the variation in gene expression levels compared to SNP genotypes. Overall, we found that adding more CpGs to the model, which were all associated to the selected expression probes of the same gene, did not significantly increase the power to explain more of the variation in gene expression (p-value < 2.2 × 10^-16^). In addition to the ADME genes, we also investigated the role of SNPs and CpG site methylation in the regulation of genes associated with diseases and liver function by querying all SNPs from the GWAS catalog (http://www.genome.gov/gwastudies/) in our list of identified eQTLs. We identified *cis*-acting SNPs and DNA methylation differences that were associated with the expression of 47 genes previously identified in different GWA studies with complex traits, including enzyme and metabolite levels as well as cardiovascular and inflammatory bowel diseases (Additional file 15).

**Table 4:** **Proportion of explained variation by SNPs and CpG sites associated with the expression of ADME genes**

Gene/Locus	Chr	SNP	CpG site	% of variation in expression explained by			
				SNP only	CpG only	SNP and CpG site	SNP and all CpG sites ^1^
*GSTT1*	22	rs9612520	cg05380919	50%	75%	78%	84%**
*CYP3A5*	7	CS015290	cg03133378	55%	7%	57%	57%
*GSTM1*	1	rs75953876	cg18938907	11%	55%	56%	61%
*GPX7*	1	rs11810754	cg11953272	48%	16%	49%	52%
*UGT1A1*	2	rs7592624	cg11811840	22%	41%	45%	47%
*SLC22A18*	11	rs413781	cg24724917	30%	15%	44%	49%*
*FMO4*	1	rs2223477	cg14981176	39%	16%	39%	39%
*GSTM3*	1	rs115636764	cg23645476	21%	20%	35%	46%**
*SLC19A1*	21	rs7867	cg27210852	22%	10%	30%	30%
*GSTO2*	10	rs11595547	cg23659134	20%	24%	28%	28%
*PON1*	7	rs854533	cg07404485	13%	23%	27%	30%
*DHRS2*	14	rs57350570	cg07125017	23%	4%	26%	26%
*GSTA4*	6	rs538920	cg22486834	14%	14%	20%	21%
*CEBPA*	19	rs80241821	cg19035908	17%	6%	20%	20%
*MGST3*	1	rs10737515	cg16553119	12%	12%	13%	13%
*DHRS7*	14	rs376391	cg18906360	12%	9%	13%	13%

*F-test p-value < 0.05; **F-test p-value < 0.005.

F-test null hypothesis: model for gene expression with the SNP and CpG site as explanatory variables and model for gene expression with the SNP and all CpG sites^1^ fit equally well with the differences being due to random chance.

SNP and all CpG sites^1^ - the CpG sites that have eQTM effects with the expression probe.

### Tissue-specificity of eQTL, meQTL and eQTMs

**Figure 4:**
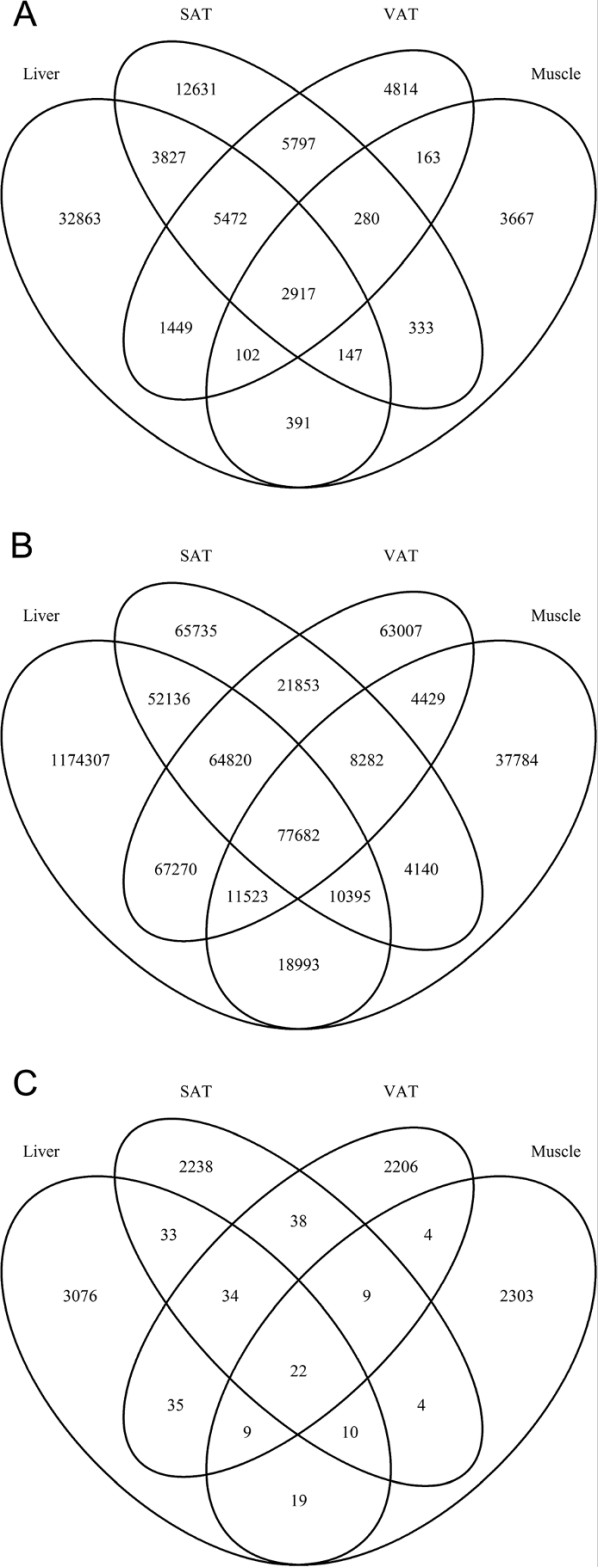
**Venn diagram of the overlap of QTLs in four tested tissues.** The number of overlapping **(A)** eQTL, **(B)** meQTLs, **(C)** eQTMs in shown for adult human liver, VAT, SAT and muscle samples.

Since we had also generated methylation and expression data for three other tissues (muscle, subcutaneous- (SAT) and visceral adipose tissue (VAT)) from the same individuals in the Dutch cohort, we could assess the tissue-specificity of the detected eQTL, meQTL and eQTM effects. We had previously compared liver eQTL with other tissues for only a limited number of samples [11], so we re-did this analysis with the new adult liver samples from the Karolinska Liver Bank. For liver eQTL, approximately 40-50% of the effects found in one tissue could also be significantly detected in another tissue (Figure 4A). We identified only a few opposite allelic effects (<1%) between the tissues (Additional file 16A and B), suggesting that if a SNP affects expression in multiple tissues, the allelic direction is mostly identical. The eQTL effects (n = 32,863) that were only present in liver and not in the other three tissues were related to genes strongly specific to liver function (p-value 5 × 10^-53^) and metabolic and catabolic processes (p-values < 5 × 10^-20^).

Contrary to the strong tissue-specificity of eQTL, meQTL were much more stable across the different tissues. On average, 70% of the meQTL are shared between at least two tissues, with over 98% of their effects having the same allelic direction (Figure 4B, Additional file 16C & D). As we had observed for eQTL, there were also a few significant meQTL that showed an opposite allelic direction between liver and the other three tissues (Additional file 17). The CpG sites of the meQTL with opposite effects were more often located outside the gene bodies (p-value 1.53 × 10^-11^), but when they were in gene bodies, they were in exons rather than introns (p-value 1.7 × 10^-90^).

As expected, we observed very strong tissue-specificity for the identified eQTMs. Only up to 4% of the eQTMs found in one tissue were also detectable with an identical effect direction in another tissue (Figure 4C, Additional file 16E).

### Genes with adult-specific functions are enriched for eQTL

We hypothesized that the expression of genes with important functions in the adult liver should be under strict genetic and epigenetic control. We thus focused on the set of probes with significantly higher and lower expression levels in adult liver compared to fetal liver (from the previous section “The transcriptome of the developing liver”), and formed a matched set of probes that were not differentially expressed between the two groups but displayed similar median expression levels and standard deviations in the adult liver samples. We observed that the expression of these adult liver-specific probes are much more likely to be affected by SNPs than the matched set of probes (1.43 times more than expected, chi-squared test p-value = 8.8 × 10^-7^). Furthermore, we observed that these probes were 1.24-fold enriched for liver-specific eQTL probes compared to a matched set of probes with eQTL in multiple tissues (chi-squared test p-value = 8.847 × 10^-6^). *Vice versa*, probes with lower expression levels in adult liver compared to fetal liver did not differ from the matched set of probes in terms of having eQTL and liver-specific eQTL effects. Furthermore, we did not observe any enrichment of meQTL in the adult liver-specific methylation probes.

## Discussion

Previous studies on the regulation of gene expression in human liver have only accounted for the effect of genetic variation in adult samples [7–11]. In this study, we investigated the developmental regulation of gene expression in human livers by comparing the expression and methylation levels of genes in adult and fetal livers. In addition, we used both genetic variants and DNA methylation differences in order to explain the variability in transcript levels observed in adult livers. Comparison of the fetal and adult liver methylomes and transcriptomes revealed that hypomethylated CpG sites and up-regulated genes were closely related to the tissue-specific functions: with fetal livers enriched for developmental and hematopoietic functions, while catabolic and metabolic processes were more prominent in adult livers. This has been described in the transcriptome of fetal livers at different stages of development in mice [23–25].

As the differences in methylation between fetal and adult livers were very large, when attempting to characterize the effects of variable methylation on gene expression levels in adults, we performed the eQTM analysis using a panel of only adult liver samples. Similarly to Gutierrez-Arcelus et al. [26], we observed both positive and negative correlations between DNA methylation and gene expression across the samples, with similar distributions across different genomic regions. Bell et al. have also observed a modest but significant excess of negative correlations between DNA methylation and variation in gene expression levels across individuals [27]. It has been reported that the role of DNA methylation appears to depend on the genomic context [28]: for example, CpG sites located near the genes and/or with a stronger correlation between the methylation and expression were more likely to display a negative correlation. Interestingly, CpG sites downstream of the expression probes displayed less negative correlations than those upstream of the probes, indicating that methylation in gene bodies is associated with active gene expression, as known from the early days of DNA methylation research [29, 30]. This paradox – in which methylation in the promoter is negatively correlated with the expression, whereas methylation in the gene body is positively correlated with expression [30] – can be explained by the fact that, in mammals, DNA methylation silences the initiation of transcription, but not transcription elongation [28].

Our eQTL mapping in adult livers revealed 751 unique genes, which were strongly liver-specific and enriched for drug metabolizing functions. Of these, 84 genes were new associations, while others have already been reported [7–10, 22]. The new associations are probably due to the larger number of samples and imputation of SNPs not present on previously used genotyping arrays, using data from the 1000 Genomes project. While we observed liver-specific associations with eQTL, the meQTL were not enriched for liver-specific functions. Furthermore, when we analyzed the SNPs that had significant effects on both methylation and expression, in most of the genes the same SNP allele had an opposite effect on gene expression compared to the methylation level, and this effect was most evident in CpG islands and shores (Additional file 10). These results show that, although there are many associations between SNPs and methylation levels, the relationships between them are not clear and do not reflect tissue-specific functions.

Inter-individual variability in ADME gene expression has been shown to affect drug efficacy, toxicity, and susceptibility to environmental toxins [31]. When we focused on the expression of ADME genes, we observed very strong *cis*-acting SNP and DNA methylation effects for 16 genes (Table 4), including members of the glutathione S-transferases (GSTs) family of phase II ADME isozymes: *GSTA4, GSTM1, GSTM3, GSTO2* and *GSTT1*; solute carrier transporters *SLC19A1* and *SLC22A18*, responsible for the transmembrane transfer of multiple drugs and endogenous compounds; and *FMO4, GPX7*, *PON1* and *UGT1A1*. GSTT1 is involved in the conjugation of a variety of compounds [32–35], while *GSTM1* functions in the detoxification of exogenous/endogenous toxins. The effects of epigenetic modifications on the expression of these genes have been reported in blood and brain tissues [36, 37]. In our study, we observed that both SNPs and DNA methylation contribute to the variability of the expression of these genes. For example, the SNP rs2739330, downstream of the *GSTT1* gene and upstream of the *DDT* gene, has been reported to be associated with gamma-glutamyl transferase levels in plasma [38]. This SNP, together with methylation levels of a nearby CpG site cg05380919, explains 78% of the variability in the expression of *GSTT1*, possibly with a stronger contribution from the methylation levels of the CpG site. Similarly, for *GSTM1* the strongest SNP only explains 11% of the variation in its expression, while methylation levels of the CpG site cg18938907 has a much stronger association with the expression of the gene, and may be responsible for up to 55% of the variation (Additional file 11). The CpG site falls within a CpG island that spans the promoter and a portion of the gene’s first intron. Interestingly, they are located near the transcription factor binding site of *TBP*, which has been shown to bind to the promoter of *GSTM1* in HepG2 cells, according to ENCODE ChIP-Seq data.

A substantial portion of the overall phenotypic variance in hepatic enzyme PON1 activity between individuals remains unexplained. Besides a variety of non-genetic factors, numerous transcription factors [39] and miRNA regulation [40], various functional PON1 polymorphisms have been shown to influence serum PON1 levels and activity [39, 41]. The SNP rs705379 has been shown to be associated with approximately 50% mean reductions in serum PON1 protein levels as well as transcript levels [41, 42]. In our study, it was interesting to see that this SNP was associated with increased methylation of nine CpG sites in its vicinity and with a lower expression of *PON1*.

Glutathione peroxidases (GPX) constitute a major antioxidative damage enzyme family [43] and are thus important in cancer therapy [44]. Not only genetic but also epigenetic mechanisms of gene regulation have been proposed for *GPX7*, while recently a CpG island was identified as a key player in regulating *GPX7* expression [45]. In total, we identified 87 SNPs in the *GPX7* gene affecting the methylation of nine CpG sites (meQTL), with six of the sites being directly implicated in quantitative differences in the gene expression. In addition, we replicated the two eQTL reported in human liver samples for *GPX7*[10], and for the first time identified their association with differences in methylation of CpG sites, which are further correlated with changes in *GPX7* expression levels. One of the expression-associated SNPs discovered in this study, rs11810754, appears to explain most of the variation in the expression levels of the gene (48%), while the CpG site with the strongest correlation with expression (cg11953272) did not add any extra information to the variability of the expression of the gene (only 1%, Table 4).

Three other tissues (muscle, SAT, VAT) were used to assess the tissue-specificity of both eQTL and meQTL effects. Our eQTL results showed that over half of the associations of SNPs with expression in one tissue could not be detected in another tissue (with identical eQTL allelic effect directions). This is similar to other studies [7, 9, 11]. In contrast, SNP-methylation correlations were much less tissue-specific than SNP-expression correlation: approximately 70% of the meQTL were also identified in any of the other tissues. To the best of our knowledge, this has not been described before, but could be due to sequence-dependent DNA methylation or the fact that genetic variation in a similarly methylated region can affect the entire region (given that we have excluded the direct effects of SNPs on methylation probes). On the other hand, we observed that DNA methylation associated with expression levels (i.e. eQTMs) are highly tissue-specific, in accordance with the fact that DNA methylation plays an important role in regulating tissue-specific gene expression. Thus, conclusions drawn from eQTL or eQTM data in one tissue cannot be extrapolated to other tissues, whereas the effect of SNPs on methylation is more likely to be detectable in an alternative tissue, for example DNA in blood, which is more readily accessible.

The greatest limitation of our study was the use of microarrays instead of massively parallel sequencing. Despite stringent filtering and remapping of expression and methylation probe sequences, we cannot rule out all technical artefacts inherent to microarray studies. Another drawback of microarrays is also their lower coverage of the genome, with the expression arrays only covering a few exons per gene, and the methylation array containing approximately 1% of the CpG sites in the human genome. Future studies using RNA and genome sequencing should be able to generate a more complete picture of the factors involved in the regulation of gene expression in human liver or other tissues.

## Conclusions

By performing a genome-wide survey of genomic and epigenomic variation and their associations with gene expression in fetal and adult human liver, we have generated a comprehensive resource for the analysis of factors involved in the regulation of hepatic gene expression. The investigation of fetal livers allowed us to explore the developmental changes in the hepatic methylome and transcriptome. Although the role of DNA methylation in different regions of the genome is still unclear, our results elucidate the coordinated effects of SNPs and methylation, as well as the tissue specificity of their effects on gene expression. This strengthens the hypothesis that knowledge of inter-individual variability, driven by genetic polymorphisms and DNA methylation marks and their interaction, is crucial for understanding the causes of differences in drug response and the etiology of diseases associated with liver function.

## Methods

The materials and methods of this study are described in detail in the Supplementary Online Methods in the Additional file 1. Briefly, our study was performed on two different cohorts, 14 fetal and 96 adult liver samples from the Karolinska Liver Bank cohort [46, 47], and 85 adult samples from the Dutch tissue cohort MORE (BBMRI obesity cohort) [11, 48]. For both datasets, the number of samples for which there is full expression, methylation and genotype data is not 100%. We therefore report the number of samples per specific analysis. DNA from the samples were genotyped using HumanOmni BeadChips (Illumina), according to the manufacturer’s instructions. We imputed both datasets using the GIANT release from the 1000 Genomes project, resulting in 5,763,069 unique SNPs, which were used in all our downstream analyses. Gene expression data was generated using HumanHT-12 BeadChips (Illumina), according to the standard protocol. Bisulfite-converted DNA samples were hybridized to Infinium HumanMethylation450 BeadChips (Illumina), following the Illumina Infinium HD Methylation protocol.

### Availability of supporting data

The data sets supporting the results of this article are available in the GEO repository, Dutch BBMRI more expression data: GSE22070; methylation data: GSE61454; Karolinska expression and methylation data: GSE61279.

## Electronic supplementary material

Additional file 1: **Supplementary materials & methods.** Detailed descriptions of the samples and methods used; information about pre-processing, QTL mapping, eQTM analysis, explained variation analysis etc. (DOCX 55 KB)

Additional file 2: **Differentially methylated genes between fetal and adult livers.** A table listing the 28,917 significant CpG sites which are differentially methylated when comparing the methylation patterns of the adult liver with the fetal liver. (XLSX 8 MB)

Additional file 3: **Average DNA methylation levels of all CpG sites on the 450K beadchip and of differentially methylated CpG sites between adult and fetal livers.** Proportion of CpG sites in (A) fetal livers and (B) adult livers with average beta-values between 0–0.25 and 0.25-0.75 and 0.75-1 grouped by CpG island regions in three different groups based on the non-significant or significant differential methylation between fetal and adult livers. (TIFF 1 MB)

Additional file 4: **Differentially expressed genes between fetal and adult liver.** A table listing the 3,284 expression probes which show significant differential expression when comparing the adult liver with the fetal liver. (XLSX 510 KB)

Additional file 5: **Differentially expressed and differentially methylated genes between fetal and adult livers.** A table listing the 1,655 genes which are both differentially expressed and differentially methylated when comparing the fetal liver with adult liver. For every gene the direction of significant expression and methylation change in fetal compared to adult liver is given. (XLSX 45 KB)

Additional file 6: **Comparison of the expression levels of transcription factors in fetal and adult livers.** A table listing the comparison of gene expression levels of transcription factors which are bioinformatically predicted to influence genes that are differentially expressed between adult and fetal livers. (XLSX 11 KB)

Additional file 7: **eQTMs identified in liver at FDR 0.05.** Information on the CpG probe, expression probe and details including significance of the effects is given in this table. (XLSX 226 KB)

Additional file 8: **eQTL identified in liver at FDR 0.05.** Information on the expression probe, SNP and details including significance of the effects is given in this table. (XLSX 7 MB)

Additional file 9: **Top 15,000 meQTL identified in liver.** Information on the methylation probe, SNP and details including significance of the effects is given in this table. Full table available upon request (too large to upload). (XLSX 20 MB)

Additional file 10: **Distribution of opposite and identical effects of a SNP on gene expression and gene methylation.** Proportion of genes with eQTL and meQTL depending on the effect of the SNP allele on gene expression compared to the methylation level grouped by CpG island regions. (TIFF 13 MB)

Additional file 11: **The contributions of SNPs and DNA methylation levels to the proportion of variation explained in gene expression levels.** A table listing the genes by the proportion of the explained variation in gene expression by either a SNP (eQTL), a CpG site (eQTM), both a SNP and a CpG site (eQTL+eQTM) or a SNP and CpG sites (eQTL+eQTMs). (XLSX 64 KB)

Additional file 12: **Unique proportion of gene expression variation explained by a SNP or a CpG site.** The figure outlines the expression variation explained uniquely by a SNP (x-axis) vs the variation explained uniquely by a CpG site (y-axis). In general the SNPs explain more gene expression variation vs. a single CpG site, however there are some exceptions. (TIFF 395 KB)

Additional file 13: **Relation between the distance from TSS and the explained variation in gene expression by a CpG site and a SNP.** Percentage of explained variation in gene expression by a CpG site or a SNP (y-axis) depending on the distance from the transcription start site of the corresponding genes (x-axis). (TIFF 2 MB)

Additional file 14: **The contributions of SNPs and DNA methylation levels to the proportion of variation explained in gene expression levels of 16 ADME genes.** Percentage of explained variation in gene expression of 16 ADME genes by a SNP (eQTL), a CpG (eQTM), both a SNP and a CpG site (eQTL+eQTM) or a SNP and CpG sites (eQTL+eQTMs). (TIFF 2 MB)

Additional file 15: **The contributions of SNPs and DNA methylation levels to the proportion of variation explained in gene expression levels of genes previously identified in GWA studies.** A table listing the genes by the amount of the explained variation in expression of genes identified in GWA studies by either a SNP (eQTL), a CpG (eQTM), both a SNP and a CpG site (eQTL+eQTM) or a SNP and CpG sites (eQTL+eQTMs). (XLSX 25 KB)

Additional file 16: **Overlapping eQTL and meQTL with the same or opposite allelic direction and eQTMs with consistent direction identified in multiple tissues.** (DOCX 15 KB)

Additional file 17: **meQTL with an opposite allelic direction between liver and the other three tissues. Illustration of a meQTL giving an opposite allelic effect in liver as compared to SAT, VAT and muscle.** The C-allele of rs9768559 is associated with decreased methylation levels at a CpG site (cg07883117) in both the liver sample sets, while in SAT, VAT and muscle the same allele is associated with increased methylation levels at the same CpG site. (TIFF 3 MB)

## Abbreviations

ADME: Absorption, distribution, metabolism, and excretion
AUC: Area under the curve
CGI: CpG islands
eQTL: Expression quantitative trait loci
eQTM: Expression quantitative trait methylation
FDR: False discovery rate
GWA: Genome-wide association
meQTL: Methylation quantitative trait loci
SAT: Subcutaneous adipose tissue
SNP: Single nucleotide polymorphism
TSS: Transcription start site
VAT: Visceral adipose tissue.
