# Supplementary material for: Genetic and epigenetic regulation of gene expression in fetal and adult human livers
Source: BMC Genomics. 2014 Oct 4;15(1):860. doi: 10.1186/1471-2164-15-860 (PMC4287518; doi:10.1186/1471-2164-15-860)
Supplement: Supplementary file 1 — Additional file 1: Supplementary materials & methods. Detailed descriptions of the samples and methods used; information about pre-processing, QTL mapping, eQTM analysis, explained variation analysis etc. (DOCX 55 KB) [file 12864_2014_6781_MOESM1_ESM.docx]

Genetic and epigenetic regulation of gene expression in fetal and adult human livers

Marc Jan Bonder, Silva Kasela et al.

## Supporting Materials & Methods

## Study subjects

Our study was performed on two different cohorts, the Karolinska Liver Bank cohort and the Dutch tissue cohort MORE (BBMRI obesity cohort). For both datasets, the number of samples for which there is full expression, methylation and genotype data is not 100%. We therefore report the number of samples per specific analysis.

### Karolinska Liver Bank samples

The Karolinska Liver Bank sample set included 96 adult and 14 fetal tissues. Two cohorts of adult tissues were collected: (1) Liver samples from 52 organ donors who had met accidental death were acquired from Karolinska University Hospital, Huddinge, Sweden, or that were purchased from the International Institute for the Advancement of Medicine (IIAM, Edison, New Jersey, USA) or from XenoTech (Lenexa, Kansas, USA). (2) An additional set of 44 liver samples was acquired from patients undergoing liver resection due to malignant tumors, most commonly from patients with metastatic colon cancers. Liver biopsies from these patients were collected from 'healthy' tissue that showed no visible pathological changes compared to the adjacent tumor. Cancer patient livers were obtained from Karolinska University Hospital and from Sahlgrenska Hospital, Gothenburg, Sweden. The adult liver characteristics have been extensively described in previous publications [1, 2]. Liver samples from 14 fetuses at gestational week 8-12 were acquired from Karolinska University Hospital, a subset of these fetal livers has been studied previously [2]. The Ethics Committees at Karolinska University Hospital approved the use of all samples for the purposes of this study.

### Dutch tissue cohort MORE

A second set of 85 samples was collected from morbidly obese Dutch individuals with a body-mass index (BMI) between 35 and 70. Tissue samples were collected from their liver, muscle, subcutaneous adipose tissue (SAT) and visceral adipose tissue (VAT), as described before [3, 4].

## Data

### Nucleic acid purification and quantification

DNA and total RNA were isolated from the Karolinska Liver Bank samples using AllPrep DNA/RNA/Protein Mini kit (QIAgen, Hilden, Germany, Cat. # 80004). DNA was quantified using Quant-iT PicoGreen dsDNA Assay kit (Invitrogen, Carlsbad, California, USA, Cat. # P11496) and SpectraMax Gemini XPS/EM Fluorescence Microplate Reader (MolecularDevices, Sunnyvale, California, USA). The RNA samples were quantified and assessed for integrity using the Agilent Bioanalyzer 2100 with the RNA 6000 Nano kit (Agilent Technologies, Santa Clara, California, USA, Cat. # 5067-1511). The RNA samples with RIN (RNA integrity number) values ≥ 8 were used for cDNA amplification. The purification of DNA and RNA, and preparation of cDNA from the Dutch tissue samples has been described before [3].

### Genotyping and imputation

DNA from 110 liver samples from the Karolinska Liver Bank were genotyped using HumanOmniExpress BeadChips (Illumina), whereas the 85 Dutch samples were analyzed using HumanOmni1-Quad BeadChips (Illumina), according to the manufacturer’s instructions. We imputed both datasets using the GIANT release from the 1000 Genomes project. For the Karolinska Liver Bank samples we used the program IMPUTE v2 to impute the genotypes of SNPs that were not present on the BeadChips.

For the Dutch samples we used the MOLGENIS compute imputation pipeline to generate our scripts and monitor the imputation. The genotypes of the Dutch samples where first aligned to the reference strand using Genotype Harmonizer. The Dutch samples where pre-phased using MACH version 1.0.18 and imputed using MINIMAC version 2012.10.3 beta [5]. The script generation and monitoring was performed using MOLGENIS compute [6].

The directly genotyped SNPs were coded as 0, 1 or 2, while for imputed SNPs we used dosage values that ranged between 0 and 2. SNPs with a lower imputation quality than 0.3 were removed from the analysis. We confined all analyses to a set of 5,763,069 unique SNPs that were present in both the Karolinska Liver Bank and Dutch samples.

### Gene expression

Starting with 300 ng of RNA, the TargetAmp™-Nano Labeling Kit for Illumina® Expression BeadChip (Epicentre, Chicago, Illinois, USA) was used to amplify and biotinylate the RNA samples from the Karolinska liver bank, according to the manufacturer’s instructions. For the Dutch samples, 200 ng of RNA was used for anti-sense RNA synthesis, amplification, and purification using the Ambion Illumina TotalPrep Amplification Kit, according to the manufacturer’s protocol (Ambion, Waltham, Massachusetts, USA). For each sample, 750 ng of biotinylated cRNA was hybridized to HumanHT-12 BeadChips (Illumina, Dutch samples on v3 arrays and Karolinska Liver Bank samples on v4 arrays), according to the standard protocol. The BeadChips were scanned within 24 h using a HiScanSQ scanner. The raw signals were exported using GenomeStudio (Illumina, USA).

### DNA methylation

From each sample, irrespective of cohort, 500 ng of genomic DNA was bisulfite modified using the EZ DNA Methylation kit (Zymo Research, Irvine, California, USA), according to the manufacturer’s recommendations for the Illumina Infinium Assay. After purification, 4 μl of each bisulfite-converted DNA sample was used for hybridization on Infinium HumanMethylation450 BeadChips, following the Illumina Infinium HD Methylation protocol. The original IDAT ﬁles were extracted from the HiScanSQ scanner.

## Data analysis

### Probe mapping and filtering

The sequences of the expression probes of the Illumina HT12-V3 and HT12-V4 BeadChips as well as the methylation probes of the Illumina HumanMethylation450 BeadChip were mapped to the latest build of the human genome (hg19) using SHRiMP [7], with transcript and genome information retrieved from Ensemble release 70.

The probes that were present on both expression platforms were used for subsequent analyses. The expression probes which mapped uniquely to the reference genome and corresponding transcripts with at least 90% identity were retained. We used this relaxed mapping criterion to be sure that there would be limited interference of possible cross-hybridization of the probes. All expression probes mapping to either the X or Y chromosomes were removed. Using this strategy, a total of 31,324 uniquely mapping expression probes remained for analysis.

The Illumina HumanMethylation450 probes were mapped using two mapping strategies. The probe sequences were mapped to the human genome reference; subsequently, we also mapped the bisulfite converted probe sequence to a similarly converted version of the human genome. All probes that mapped uniquely on both genomes with a minimal sequencing identity of 90% were retained. Again, all probes on the X and Y chromosomes were removed. Finally, all probes containing SNPs up to three bases from the single base extension site were discarded, as these have been shown to affect the primer extension [8]. Using this strategy, we included a total of 366,306 methylation probes for subsequent analyses. Using information from the UCSC genome browser we linked probes to known CpG island (CGI) regions. Probes inside a known CGI were marked as an “Island” probe, probes within a region of maximally 2 kb upstream or downstream of a CGI were marked as North-Shore or South-Shore, respectively, and probes between 2 to 4 kb upstream or downstream of a CGI were marked North-Shelf or South-Shelf, respectively. Using the transcript information of the Ensemble database we linked CpG sites to the closest gene and annotated their location within the gene.

### Expression normalization

The raw expression intensities from the two liver datasets and three other tissues were quantile normalized and log_2_ transformed. Using MixupMapper [9] we corrected for mix-ups in the expression data. We further applied a principal component analysis (PCA) on the expression correlation matrix, separately for each tissue type, to correct for physiological or environmental variation (e.g., tissue type and phenotype difference) as well as systematic experimental variation (e.g. batch and technical effect). In order to target the difference in the genetic variation of expression among tissues, we removed the global variation in expression among tissues by using the residual expression for each probe in each tissue after first removing the optimal number of principal components per tissue type. This optimum was determined based on the maximum number of *cis*-eQTLs. Optimal PCA correction is described in [10]. The optimal number of PCs to remove was ten in liver, fifteen in SAT, twenty in VAT, and five in the muscle samples. After sample preprocessing, 14 fetal and 92 adult livers from the Karolinska liver bank, and 80 liver, 67 muscle, 85 SAT, and 80 VAT tissue samples from the Dutch cohort were included for subsequent analyses.

### Methylation normalization

Data pre-processing and quality control analyses were performed in R using the Bioconductor package minfi [11]. ‘Raw’ pre-processing was used to convert the intensities from the red and the green channels into methylated and unmethylated signals. Beta values were computed using Illumina’s formula [beta = M/(M+U+100)]. The difference in the distribution of beta values for type I and type II probes was corrected using “SWAN” [12], a normalization method to deal with systematic changes between type I and type II probes. Detection p-values were obtained for every CpG probe in every sample. Failed positions were defined as signal levels lower than background from both the methylated and unmethylated channels. After SWAN normalization we transformed the Beta-values to M-values using: M = log_2_(beta/1-beta). The M-values were quantile normalized and PCs were calculated as described for the expression data. The optimal number of PCs to remove, which was calculated using the same procedure as for the transcriptomic analysis, was fourteen in liver, six in SAT, six in VAT and six in the muscle samples. After sample preprocessing, the total number of samples with methylation data was 14 fetal and 96 adult livers from the Karolinska Liver Bank, and 67 liver, 60 muscle, 71 SAT, and 71 VAT tissue samples from the Dutch cohort.

### Differential expression analysis

Differential expression analysis between fetal and adult samples was conducted on the samples from the Karolinska Liver Bank. Information about detection p-values was used to estimate the present count of each probe. Probes with detectable expression in < 10% of samples (detection p-value < 0.05) were discarded. Next, the expression data was log2 transformed and normalized using quantile normalization with the lumi package in R. The sequences of the expression probes were mapped to the human genome reference (build hg19). The expression probes which mapped uniquely to the reference genome and corresponding transcripts with at least 90% identity were retained. All expression probes mapping to either the X or Y chromosomes were discarded, which yielded 20,715 probes for analysis. The *limma* [13] package was used to fit linear models predicting expression at each probe, adjusted for sex, chip and position on chip, using the empirical Bayes method implemented in the package. The criteria for significance were determined by absolute log_2_-fold change > 1 and Benjamin-Hochberg false discover rate (FDR) < 5%. The gender of the fetal samples was determined based on *XIST* gene expression levels.

### Differential methylation analysis

The SWAN normalized dataset was used for the analysis of differential methylation between fetal and adult samples from the Karolinska Liver Bank. Beta-values were quantile normalized and transformed into M-values. In addition to removing CpG sites as described in “Probe mapping and filtering”, CpG sites with non-significant detection p-values (> 0.01) in more than 10% of the samples were discarded, resulting in 366,074 CpG sites for analysis. Linear regression models for each of the CpG sites were fitted and adjusted for sex, chip and position on chip. The criteria for significance were determined by absolute difference in mean Beta-values > 0.2 and Benjamin-Hochberg FDR < 5%. The gender of the fetal samples was confirmed based on DNA methylation patterns of X and Y chromosome.

### Quantitative Trait Loci (QTL) analysis

In order to determine the effect of nearby SNPs on expression (*cis*-eQTLs) and methylation levels (*cis*-meQTLs), the analysis was confined to those probe-SNP combinations for which the distance from the probe midpoint to the SNP genomic location was up to 250 kb. For each probe-SNP pair, we used the Spearman correlation coefficient to detect association between SNPs and the variations of the gene expression in liver, SAT, VAT and muscle. We calculated the Spearman correlation coefficient and corresponding p-values and subsequently transformed this into a Z-score. To maximize the power of QTL detection in liver, we performed a meta-analysis for the two independent liver datasets. An overall, joint p-value was calculated using a weighted (square root of the dataset sample number) Z-method. A comprehensive overview of this method has been described previously [14].

To correct for multiple testing, we controlled the FDR at 5%. The distribution of observed p-values was used to calculate the FDR, by comparison with the distribution obtained from permuting expression phenotypes relative to genotypes 50 times. We filtered both the identified *cis-*eQTLs and *cis-*meQTLs effects for false positive effects caused by LD between the QTL-SNP and SNPs in the QTL probe. If we observed LD between these SNPs, higher than R^2^ 0.2, we removed the QTL effect. We determined these effects in the two datasets which were used for the QTL analysis, as well as using data from the HapMap3 and 1000 Genomes projects, in order to be as strict as possible in marking a QTL as true positive.

### Expression quantitative trait methylation (eQTM) analysis

To identify methylation levels correlating with the expression of nearby genes (*cis*-eQTMs) we adapted the idea of multivariate permutation tests [15]. Firstly, we selected one expression probe and all methylation CpG sites within 250 kb of the probe. Secondly, we calculated a Spearman correlation coefficient for each probe-CpG pair (observed correlation coefficients). Thirdly, we randomly shuffled the sample labels of the expression probe and again calculated the Spearman ranked correlation coefficient between the permuted expression probe and nearby methylation CpG sites. Only the largest absolute correlation coefficient of the given set was saved. The rearrangement procedure was conducted 5000 times and the largest absolute coefficient was saved each time. Finally, to estimate the significance of the eQTM effect, a permutation p-value was computed as the probability to observe a random absolute correlation coefficient as extreme as or more extreme than the given observed coefficient. Then the next expression probe was selected and the described process was repeated for each probe.

### Explained variation in gene expression

In order to estimate how much of the variation in gene expression we can explain by SNPs and methylation, we fitted four different linear models:

1. $y_{i} \sim\beta_{0}+ \beta_{1}*x_{i}+e_{i} ,$
2. $y_{i} \sim\beta_{0}+ \beta_{1}*z_{i}+e_{i} ,$
3. $y_{i} \sim\beta_{0}+ \beta_{1}*x_{i}+\beta_{2}*z_{i}+e_{i} ,$
4. $y_{i} \sim\beta_{0}+ \beta_{1}*x_{i}+\beta_{2}*z_{i1}+\cdots+\beta_{k+2}*z_{ik}+e_{i} ,$

where $y_{i}$ is the gene expression levels of the probe *i*, $x_{i}$ is the corresponding imputed eQTL SNP dosage values, $z_{i}$ is the methylation levels of the corresponding eQTM CpG site, *k* is the number of eQTM effects of probe *i,* $\beta_{0}$ is the intercept term, $\beta_{1}, \ldots,\beta_{k+2}$ are the regression coefficients and $e_{i}$ is the error term. For each model the coefficient of determination (R^2^) was interpreted as the proportion of total variation of the outcome variable that can be explained by the explanatory variables, i.e. the proportion of variation in gene expression explained by a SNP (model 1) or by a CpG site (model 2) or by a SNP and a CpG site(s) (model 3 and 4).

Thereby, for every gene expression probe one SNP and one CpG pair, which explained most of the variation in gene expression, were chosen as the best model for a particular gene. We calculated semipartial correlations which represent the correlation between the outcome and an explanatory variable that has been residualized with respect to the other variables in the model to fully deconstruct the explained variance components. Each squared semipartial correlation coefficient represents the proportion of explained variance that is uniquely associated with the explanatory variable. Hence, the sum of the squared semipartial correlations can be subtracted from the overall R^2^ to determine the amount of common variance in the equation shared by multiple explanatory variables with the outcome. To further determine if we could achieve an even higher explained variance by adding all the CpG sites which have eQTM effects with the particular probe to the model (model 4), an F-test was used to compare the best and the fourth model, i.e. the null hypothesis tested was that both models fit equally well and the differences are due to random chance.

### Gene ontology analysis

Gene ontology analysis for all methylation probes based analysis were performed using the Genomic Regions Enrichment of Annotations Tool (GREAT; [16] using default settings. All gene expression pathway analyses were performed with the Gene Network pathway analysis tool [17]. The lists of gene symbols that appeared to be differentially expressed between tissues were submitted to Gene Network, and enriched GO terms and Transcription factors were extracted, using Wilcoxon-Mann-Whitney U test.

## References

1. Kacevska M, Ivanov M, Wyss A, Kasela S, Milani L, Rane A, Ingelman-Sundberg M: **DNA methylation dynamics in the hepatic CYP3A4 gene promoter.** *Biochimie* 2012, **94:**2338-2344.

2. Ivanov M, Kals M, Kacevska M, Barragan I, Kasuga K, Rane A, Metspalu A, Milani L, Ingelman-Sundberg M: **Ontogeny, distribution and potential roles of 5-hydroxymethylcytosine in human liver function.** *Genome Biol* 2013, **14:**R83.

3. Fu J, Wolfs MG, Deelen P, Westra HJ, Fehrmann RS, Te Meerman GJ, Buurman WA, Rensen SS, Groen HJ, Weersma RK, et al: **Unraveling the regulatory mechanisms underlying tissue-dependent genetic variation of gene expression.** *PLoS Genet* 2012, **8:**e1002431.

4. Wolfs MG, Rensen SS, Bruin-Van Dijk EJ, Verdam FJ, Greve JW, Sanjabi B, Bruinenberg M, Wijmenga C, van Haeften TW, Buurman WA, et al: **Co-expressed immune and metabolic genes in visceral and subcutaneous adipose tissue from severely obese individuals are associated with plasma HDL and glucose levels: a microarray study.** *BMC Med Genomics* 2010, **3:**34.

5. Howie B, Fuchsberger C, Stephens M, Marchini J, Abecasis GR: **Fast and accurate genotype imputation in genome-wide association studies through pre-phasing.** *Nat Genet* 2012, **44:**955-959.

6. Byelas H, Martijn D, Pieter N, Freerk vD, Alexandros K, *P*atrick D, Morris S: **Scaling Bio-Analyses from Computational Clusters to Grids.** In *International Workshop on Science Gateways; Switzerland*. Edited by Kiss T. 2013

7. Rumble SM, Lacroute P, Dalca AV, Fiume M, Sidow A, Brudno M: **SHRiMP: accurate mapping of short color-space reads.** *PLoS Comput Biol* 2009, **5:**e1000386.

8. Nordlund J, Bäcklin CL, Wahlberg P, Busche S, Berglund EC, Eloranta ML, Flaegstad T, Forestier E, Frost BM, Harila-Saari A, et al: **Genome-wide signatures of differential DNA methylation in pediatric acute lymphoblastic leukemia.** *Genome Biol* 2013, **14:**r105.

9. Westra HJ, Jansen RC, Fehrmann RS, te Meerman GJ, van Heel D, Wijmenga C, Franke L: **MixupMapper: correcting sample mix-ups in genome-wide datasets increases power to detect small genetic effects.** *Bioinformatics* 2011, **27:**2104-2111.

10. Fehrmann RS, Jansen RC, Veldink JH, Westra HJ, Arends D, Bonder MJ, Fu J, Deelen P, Groen HJ, Smolonska A, et al: **Trans-eQTLs reveal that independent genetic variants associated with a complex phenotype converge on intermediate genes, with a major role for the HLA.** *PLoS Genet* 2011, **7:**e1002197.

11. Aryee MJ, Jaffe AE, Corrada-Bravo H, Ladd-Acosta C, Feinberg AP, Hansen KD, Irizarry RA: **Minfi: a flexible and comprehensive Bioconductor package for the analysis of Infinium DNA methylation microarrays.** *Bioinformatics* 2014.

12. Maksimovic J, Gordon L, Oshlack A: **SWAN: Subset-quantile within array normalization for illumina infinium HumanMethylation450 BeadChips.** *Genome Biol* 2012, **13:**R44.

13. Smyth GK: **Linear models and empirical bayes methods for assessing differential expression in microarray experiments.** *Stat Appl Genet Mol Biol* 2004, **3:**Article3.

14. Whitlock MC: **Combining probability from independent tests: the weighted Z-method is superior to Fisher's approach.** *J Evol Biol* 2005, **18:**1368-1373.

15. Blackford JU, Salomon RM, Waller NG: **Detecting change in biological rhythms: a multivariate permutation test approach to Fourier-transformed data.** *Chronobiol Int* 2009, **26:**258-281.

16. McLean CY, Bristor D, Hiller M, Clarke SL, Schaar BT, Lowe CB, Wenger AM, Bejerano G: **GREAT improves functional interpretation of cis-regulatory regions.** *Nat Biotechnol* 2010, **28:**495-501.

17. Cvejic A, Haer-Wigman L, Stephens JC, Kostadima M, Smethurst PA, Frontini M, van den Akker E, Bertone P, Bielczyk-Maczyńska E, Farrow S, et al: **SMIM1 underlies the Vel blood group and influences red blood cell traits.** *Nat Genet* 2013, **45:**542-545.
