# Supplementary figures and images for: Genetic and epigenetic regulation of gene expression in fetal and adult human livers
Source: BMC Genomics. 2014 Oct 4;15(1):860. doi: 10.1186/1471-2164-15-860 (PMC4287518; doi:10.1186/1471-2164-15-860)

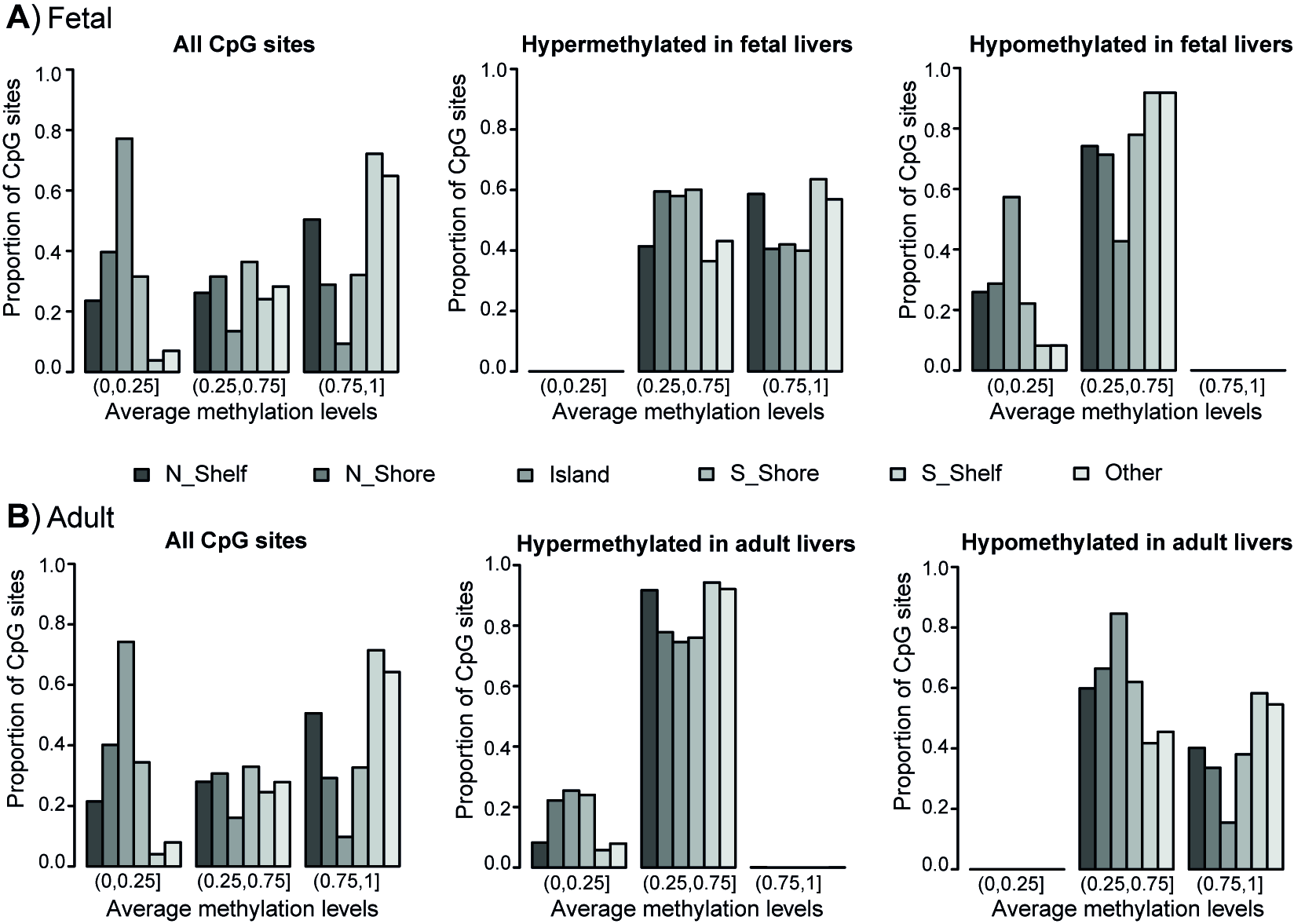

Supplement: Supplementary file 3 — Additional file 3: Average DNA methylation levels of all CpG sites on the 450K beadchip and of differentially methylated CpG sites between adult and fetal livers. Proportion of CpG sites in (A) fetal livers and (B) adult livers with average beta-values between 0–0.25 and 0.25-0.75 and 0.75-1 grouped by CpG island regions in three different groups based on the non-significant or significant differential methylation between fetal and adult livers. (TIFF 1 MB) [file 12864_2014_6781_MOESM3_ESM.tiff]

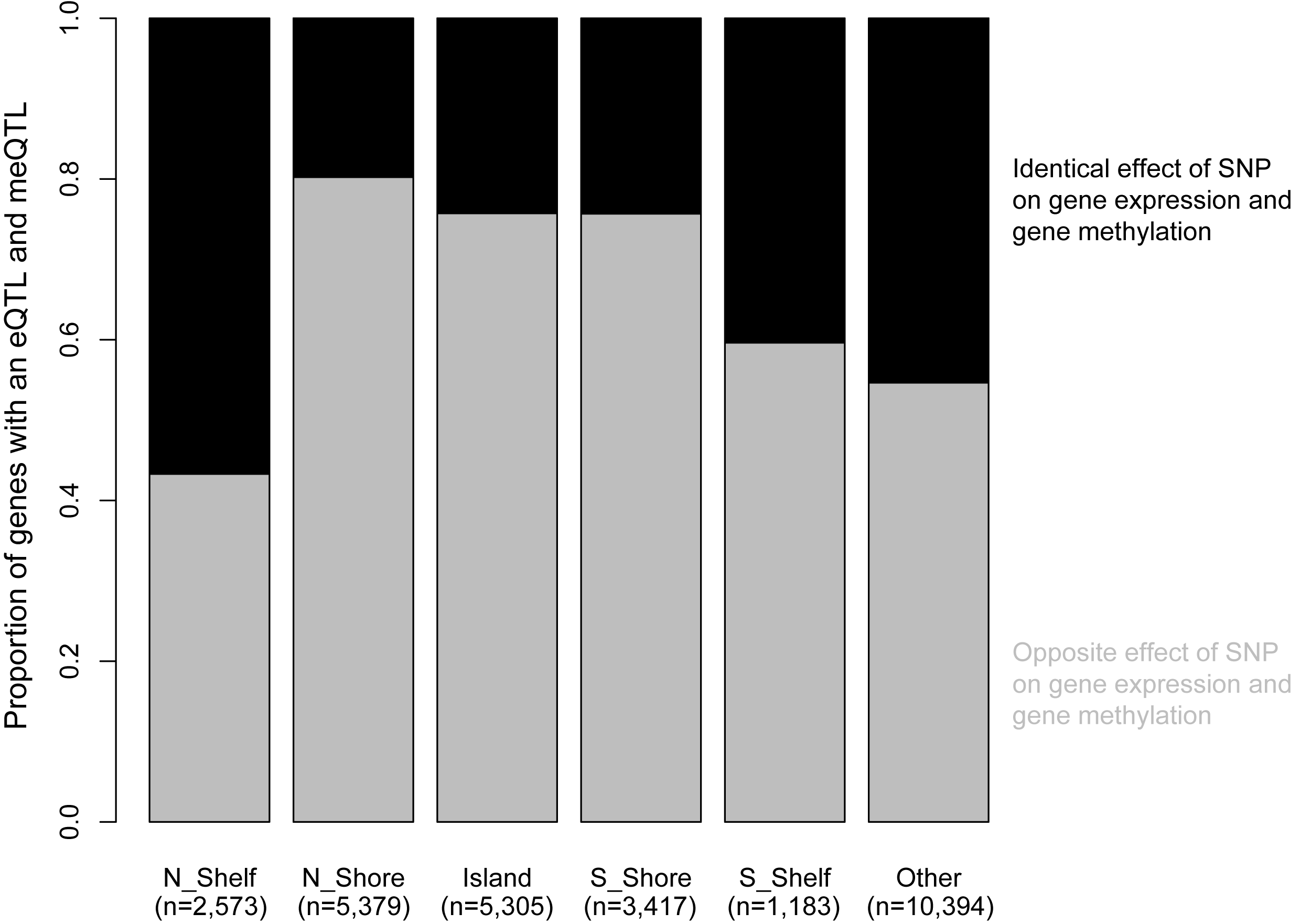

Supplement: Supplementary file 10 — Additional file 10: Distribution of opposite and identical effects of a SNP on gene expression and gene methylation. Proportion of genes with eQTL and meQTL depending on the effect of the SNP allele on gene expression compared to the methylation level grouped by CpG island regions. (TIFF 13 MB) [file 12864_2014_6781_MOESM10_ESM.tiff]

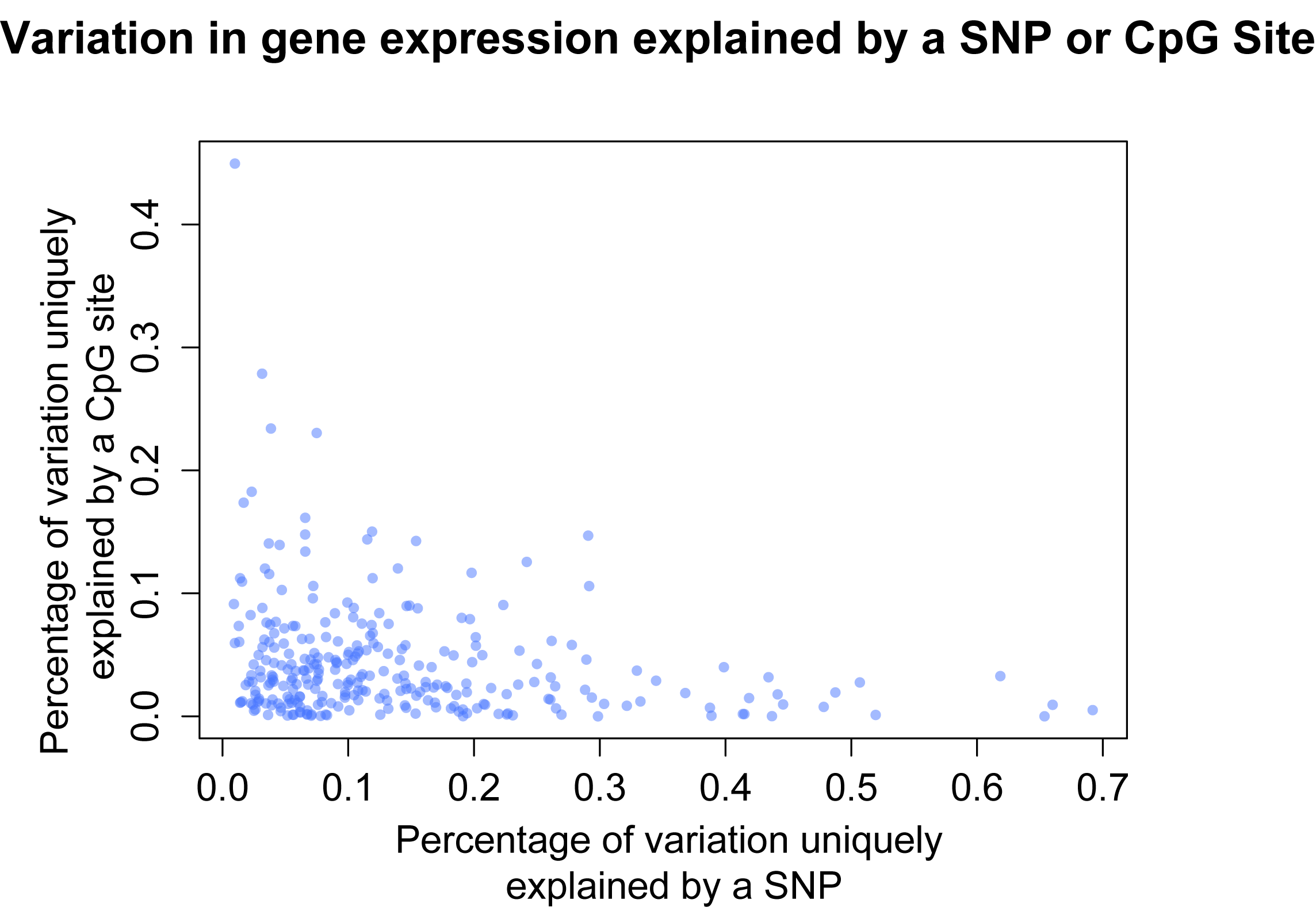

Supplement: Supplementary file 12 — Additional file 12: Unique proportion of gene expression variation explained by a SNP or a CpG site. The figure outlines the expression variation explained uniquely by a SNP (x-axis) vs the variation explained uniquely by a CpG site (y-axis). In general the SNPs explain more gene expression variation vs. a single CpG site, however there are some exceptions. (TIFF 395 KB) [file 12864_2014_6781_MOESM12_ESM.tiff]

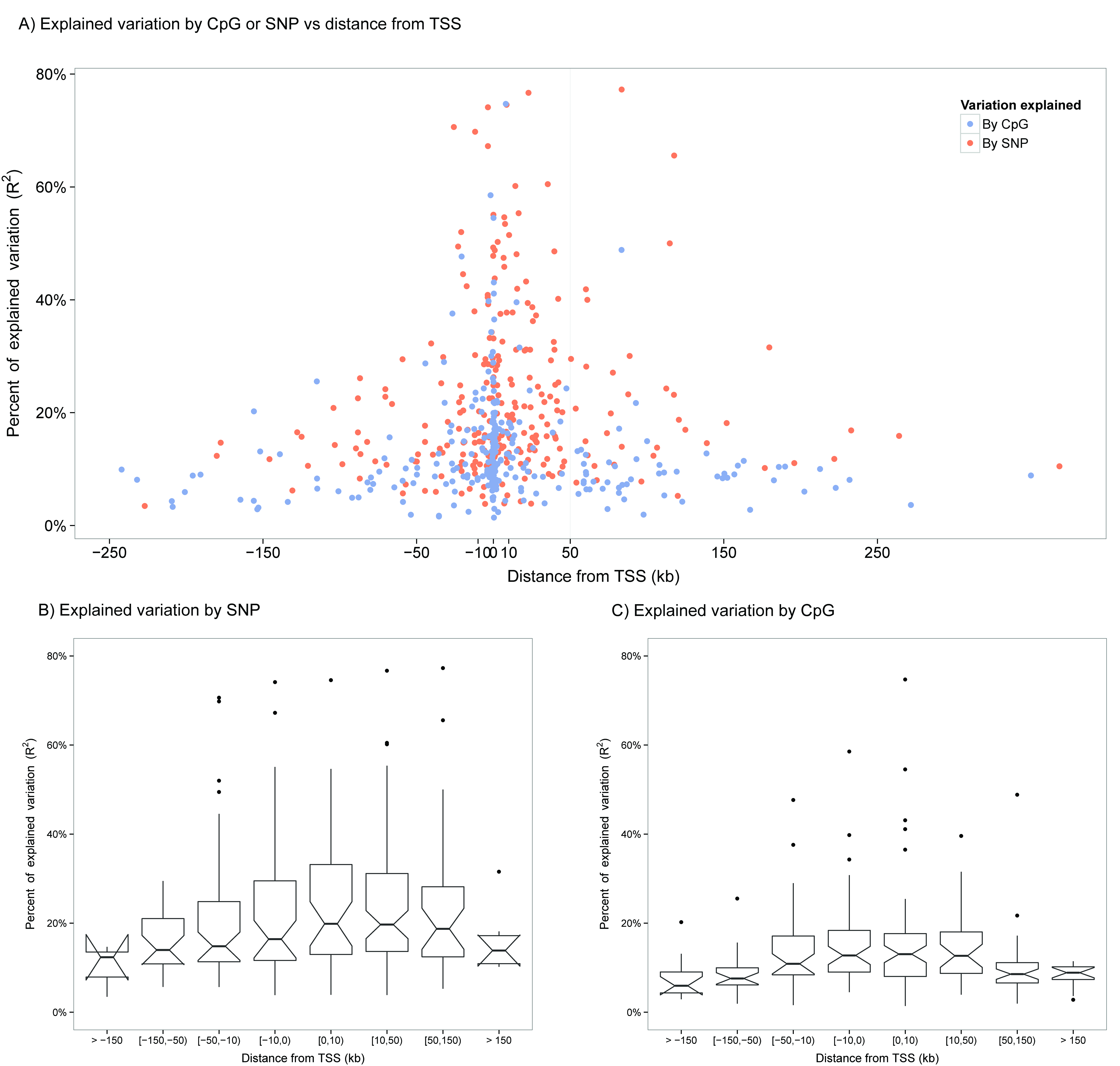

Supplement: Supplementary file 13 — Additional file 13: Relation between the distance from TSS and the explained variation in gene expression by a CpG site and a SNP. Percentage of explained variation in gene expression by a CpG site or a SNP (y-axis) depending on the distance from the transcription start site of the corresponding genes (x-axis). (TIFF 2 MB) [file 12864_2014_6781_MOESM13_ESM.tiff]

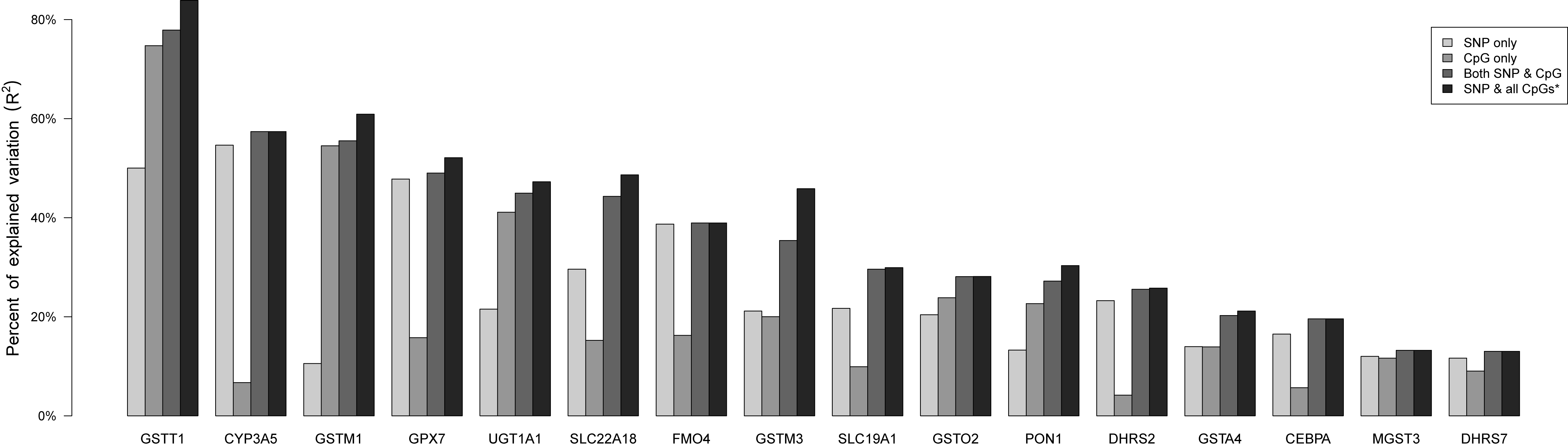

Supplement: Supplementary file 14 — Additional file 14: The contributions of SNPs and DNA methylation levels to the proportion of variation explained in gene expression levels of 16 ADME genes. Percentage of explained variation in gene expression of 16 ADME genes by a SNP (eQTL), a CpG (eQTM), both a SNP and a CpG site (eQTL+eQTM) or a SNP and CpG sites (eQTL+eQTMs). (TIFF 2 MB) [file 12864_2014_6781_MOESM14_ESM.tiff]

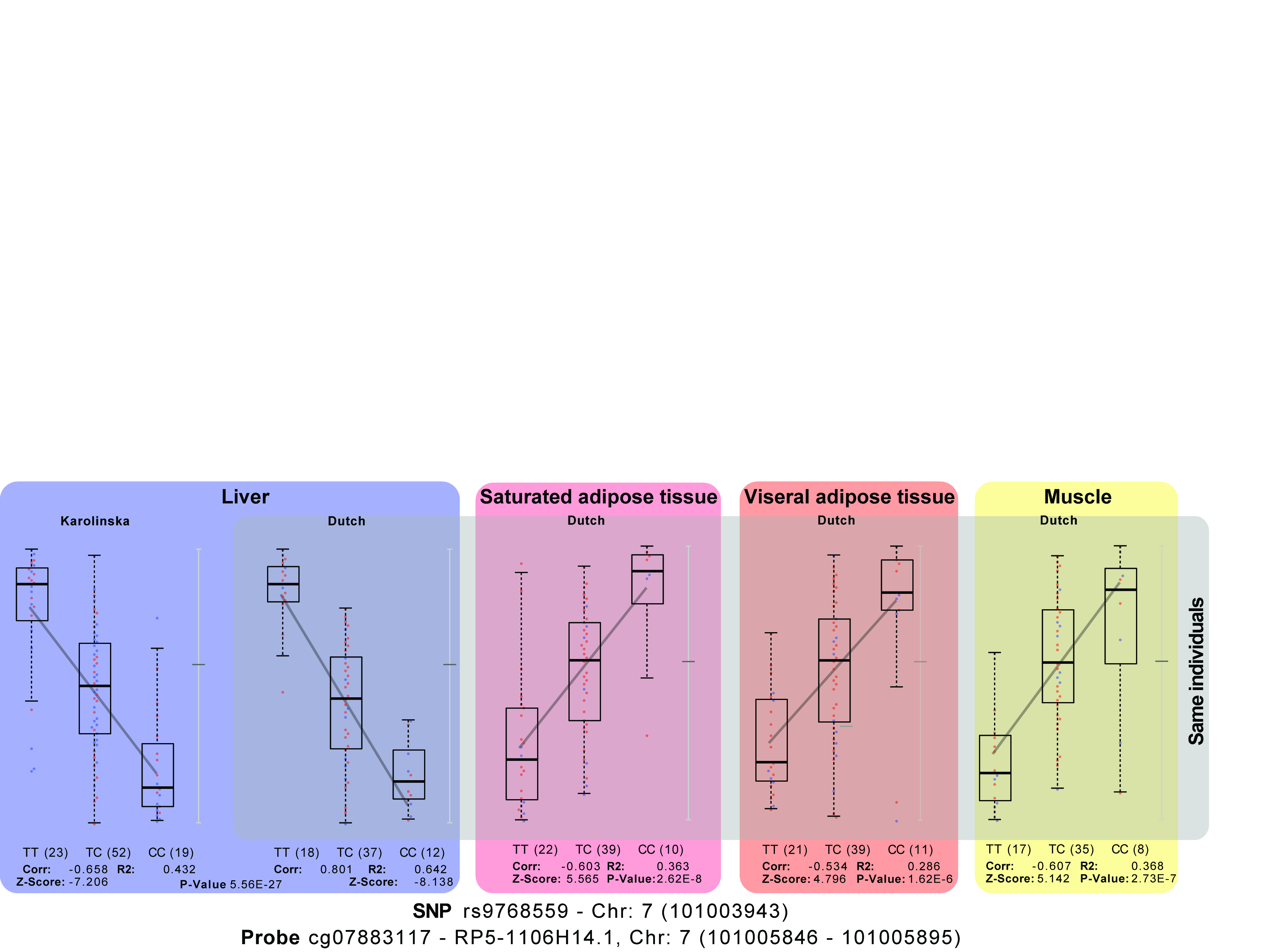

Supplement: Supplementary file 17 — Additional file 17: meQTL with an opposite allelic direction between liver and the other three tissues. Illustration of a meQTL giving an opposite allelic effect in liver as compared to SAT, VAT and muscle. The C-allele of rs9768559 is associated with decreased methylation levels at a CpG site (cg07883117) in both the liver sample sets, while in SAT, VAT and muscle the same allele is associated with increased methylation levels at the same CpG site. (TIFF 3 MB) [file 12864_2014_6781_MOESM17_ESM.tiff]
