# Supplementary material for: Genetic and epigenetic regulation of gene expression in fetal and adult human livers
Source: BMC Genomics. 2014 Oct 4;15(1):860. doi: 10.1186/1471-2164-15-860 (PMC4287518; doi:10.1186/1471-2164-15-860)
Supplement: Supplementary file 16 — Additional file 16: Overlapping eQTL and meQTL with the same or opposite allelic direction and eQTMs with consistent direction identified in multiple tissues. (DOCX 15 KB) [file 12864_2014_6781_MOESM16_ESM.docx]

(A) Number of overlapping eQTLs comparing two tissues with consistent allelic direction. In grey the number of eQTLs per tissue is given.

|  | | **Compared to** | | | |
| --- | --- | --- | --- | --- | --- |
| **Tissue** | **NR eQTLs** | **Liver** | **SAT** | **VAT** | **Muscle** |
| **Liver** | 47,168 | - | 12,363 (26.2%) | 9,940 (21.1%) | 3,557 (7.5%) |
| **SAT** | 31,404 | 12,363 (39.4%) | - | 14,466 (46.1%) | 3,667 (11.7%) |
| **VAT** | 20,994 | 9,940 (47.4%) | 14,466 (69.1%) | - | 3,462 (16.4%) |
| **Muscle** | 8,000 | 3,557 (44.6%) | 3,667 (45.9%) | 3,462 (43.3%) | - |

(B) Number of overlapping eQTL effects which have opposite allelic direction.

|  | **Compared to** | | |
| --- | --- | --- | --- |
| **Tissue** | **SAT** | **VAT** | **Muscle** |
| **Liver** | 7 (0.1%) | 36 (0.4%) | 4 (0.1%) |
| **SAT** |  | 0 (0.0%) | 0 (0.0%) |
| **VAT** |  |  | 0 (0.0%) |

(C) Number of overlapping meQTLs comparing two tissues with consistent direction. In grey the number of meQTLs per tissue is given.

|  | | **Compared to** | | | |
| --- | --- | --- | --- | --- | --- |
| **Tissue** | **NR meQTLs** | **Liver** | **SAT** | **VAT** | **Muscle** |
| Liver | 1,477,126 | - | 205,033 (13.7%) | 221,295 (14.9%) | 118,593 (7.9%) |
| SAT | 305,043 | 205,033 (67.21%) | - | 172,637 (54.1%) | 100,499 (32.9%) |
| VAT | 318,866 | 221,295 (69.4%) | 172,637 (56.6%) | - | 101,916 (31.9%) |
| Muscle | 173,228 | 118,593 (68.5%) | 100,499 (58.1%) | 101,916 (58.9%) | - |

(D) Number of overlapping meQTL effects which have opposite allelic direction.

|  | **Compared to** | | |
| --- | --- | --- | --- |
| **Tissue** | **SAT** | **VAT** | **Muscle** |
| Liver | 1,471 (0.72%) | 1,009 (0.46%) | 1,920 (1.62%) |
| SAT |  | 0 (0.0%) | 53 (0.05%) |
| VAT |  |  | 23 (0.02%) |

(E) Number of overlapping eQTMs with consistent direction across tissues. In grey the number of eQTMs per tissue is given

|  | | **Compared to** | | | |
| --- | --- | --- | --- | --- | --- |
| **Tissue** | **NR eQTMs** | **Liver** | **SAT** | **VAT** | **Muscle** |
| **Liver** | 3,238 | - | 98 (3.03%) | 99 (3.06%) | 58 (1.79%) |
|  |  |  |  |  |  |
| **SAT** | 2,388 | 98 (4.10%) | - | 99 (4.15%) | 45 (1.88%) |
| **VAT** | 2,357 | 99 (4.20%) | 99 (4.20%) | - | 42 (1.78%) |
| **Muscle** | 2,380 | 58 (2.44%) | 45 (1.89%) | 42 (1.76%) | - |
